# The Medical Student’s Manual of Chemistry

**Published:** 1907-03

**Authors:** 


**The Medical Student's Manual of Chemistry.** By **R. A. Witthaus,** M.D., Professor of Chemistry, Physics and Toxicology in Cornell University. Sixth edition. Octavo, pp. 820. New York: William Wood & Co. 1906. (Price, $4.00).

In the new edition of this excellent chemistry the section of chemical physics and general chemistry has been rewritten insofar as the latter applies to chemical physics. Inorganic chemistry has been reduced to its smallest compass, for the author very properly believes that the object of chemical teaching should not be to cram the student mind with a store of isolated facts, but rather to train him to reason out chemical problems for himself. Organic chemistry has been rewritten in order to conform to the latest information upon the relationship of substances.

Physiologic chemistry has been amplified and much space is given to the consideration of the proteins and other substances of unknown constitution, more particularly the composition of the tissues of the fluids of the body and the chemical processes occurring therein. The questions which are still unsolved or yet under discussion are either very briefly referred to or passed over without comment. The necessity for keeping pace with the rapid growth of chemical science has of course, made the book much larger than its predecessors. Altogether it is one of the most complete and comprehensive works on modern chemistry now before the profession.

